# A Key mRNA-miRNA-lncRNA Competing Endogenous RNA Triple Sub-network Linked to Diagnosis and Prognosis of Hepatocellular Carcinoma

**DOI:** 10.3389/fonc.2020.00340

**Published:** 2020-03-17

**Authors:** Junjie Zhang, Weiyang Lou

**Affiliations:** ^1^Department of Hepatobiliary Surgery, The First People's Hospital of Fuyang Hangzhou, Hangzhou, China; ^2^Department of Breast Surgery, The First Affiliated Hospital of Zhejiang University, College of Medicine, Zhejiang University, Hangzhou, China

**Keywords:** hepatocellular carcinoma, competing endogenous RNA (ceRNA), diagnosis, prognosis, log non-coding RNA (lncRNA), microRNA (miRNA)

## Abstract

Growing evidence has illustrated critical roles of competing endogenous RNA (ceRNA) regulatory network in human cancers including hepatocellular carcinoma. In this study, we aimed to find promising diagnostic and prognostic biomarkers for patients with hepatocellular carcinoma. Three novel unfavorable prognosis-associated genes (CELSR3, GPSM2, and CHEK1) was first identified. We also demonstrated that these genes were significantly upregulated in hepatocellular carcinoma cell lines and tissues. Next, 154 potential miRNAs of CELSR3, GPSM2, and CHEK1 were predicted. CHEK1-hsa-mir-195-5p/hsa-mir-497-5p and GPSM2-hsa-mir-122-5p axes were defined as two key pathways in carcinogenesis of hepatocellular carcinoma by combination of *in silico* analysis and experimental validation. Subsequently, lncRNAs binding to hsa-mir-195-5p, hsa-mir-497-5p, and hsa-mir-122-5p were predicted via starBase and miRNet databases. After performing expression analysis and survival analysis for these predicted lncRNAs, we showed that nine lncRNAs (SNHG1, SNHG12, LINC00511, HCG18, FGD5-AS1, CERS6-AS1, NUTM2A-AS1, SNHG16, and ASB16-AS1) were markedly increased in hepatocellular carcinoma and their upregulation indicated poor prognosis. Moreover, a similar mRNA-miRNA-lncRNA analysis for six “known” genes (CLEC3B, DNASE1L3, PTTG1, KIF2C, XPO5, and UBE2S) was performed. Subsequently, a comprehensive mRNA-miRNA-lncRNA triple ceRNA network linked to prognosis of patients with hepatocellular carcinoma was established. Moreover, all RNAs in this network exhibited significantly diagnostic values for patients with hepatocellular carcinoma. In summary, the current study constructed a mRNA-miRNA-lncRNA ceRNA network associated with diagnosis and prognosis of hepatocellular carcinoma.

## Introduction

Hepatocellular carcinoma is one of the most common types of cancer, with nearly 600,000 newly-diagnosed cases of patients with hepatocellular carcinoma (1–3).

Although great advances in the therapeutic approaches have been achieved in the past decades, the prognosis of patients with hepatocellular carcinoma remains dismal, with five-year survival rates less than 20% (4). Most of patients diagnosed at advanced stage of hepatocellular carcinoma and high incidence of recurrence and metastasis after curative therapies may account for this poor prognosis (5). Therefore, exploring detailed mechanisms of pathogenesis of hepatocellular carcinoma and identifying promising diagnostic and prognostic biomarkers of hepatocellular carcinoma may be helpful for providing effective therapeutic targets and improving patients' outcome. A hypothesis, namely competing endogenous RNA (ceRNA), that non-coding RNA (ncRNA), including long non-coding RNA (lncRNA), can cross-talk with messenger RNA (mRNA) by competitively binding to shared miRNAs was proposed by Salmena et al. (6). In recent years, a variety of studies regarding roles of competing endogenous RNA (ceRNA) regulatory network in human cancers have been launched and lots of attractive findings have been obtained. For example, Li et al. (7) identified prognostic signatures associated with long-term overall survival of thyroid cancer patients based on a competing endogenous RNA network; Wang et al. (8) found some prognostic markers for glioblastoma by ceRNA network analysis; Wang et al. (9) constructed a mRNA-miRNA-lncRNA competing endogenous RNA triple sub-network associated with prognosis of pancreatic cancer. However, current knowledge about ceRNA regulatory network in hepatocellular carcinoma remains extremely limited and need to be further probed. In this study, by employing “mRNA-miRNA-lncRNA” order pattern instead of “lncRNA-miRNA-mRNA” order pattern, we constructed a comprehensive ceRNA sub-network, in which all RNAs possess significant diagnostic and prognostic values for patients with hepatocellular carcinoma.

## Materials and Methods

### GEPIA Database Analysis

GEPIA (Gene Expression Profiling Interactive Analysis, http://gepia.cancer-pku.cn/detail.php) is a newly developed interactive web server for analyzing the RNA sequencing expression data from The Cancer Genome Atlas (TCGA) and the Genotype-Tissue Expression (GTEx) projects (10). GEPIA was employed to obtain the genes most associated with overall survival and disease-free survival of patients with hepatocellular carcinoma. Logrank *P* < 0.05 was considered as statistically significant. GEPIA was also used to perform expression analysis and survival analysis of lncRNA. The expression correlation between potential mRNA-lncRNA pairs was also determined by GEPIA. For expression analysis, *P* < 0.05 was considered as statistically significant. For correlation analysis, *R* > 0.1 and *P* < 0.05 were set as the criteria for identifying significant mRNA-lncRNA interactive pairs.

### UALCAN Database Analysis

mRNA expression slevels of three candidate genes (CELSR3, GPSM2, and CHEK1) in hepatocellular carcinoma were further detected using UALCAN database (http://ualcan.path.uab.edu/index.html), which is a comprehensive, user-friendly, and interactive web resource for analyzing cancer data (11). *P* < 0.05 was considered as statistically significant.

### Oncomine Database Analysis

Oncomine (https://www.oncomine.org/) is a cancer microarray database and an integrated data-mining platform (12). In this study, Oncomine was utilized to analyze mRNA expression of CELSR2, GPSM2, and CHEK1 in hepatocellular carcinoma by conducting a meta-analysis of datasets as we previously described (13). *P* < 0.05 and |fold change| > 1.5 were set as the thresholds for selecting included datasets.

### HumanProteinAtlas Database Analysis

HumanProteinAtlas (https://www.proteinatlas.org/) is a Swedish-based database, which was initiated in 2003 with the aim to map all the human proteins in cells, tissues and organs (14–16). CELSR2, GPSM2, and CHEK1 protein expression levels in hepatocellular carcinoma were assessed using HumanProteinAtlas database.

### miRNet Database Analysis

miRNet (http://www.mirnet.ca/), an easy-to-use online tool for miRNA-associated studies, was used to predict potential miRNAs binding to mRNAs (17, 18). Besides, it was also employed to predict potential lncRNAs binding to miRNAs. mRNA-miRNA and miRNA-lncRNA regulatory networks were subsequently established by Cytoscape software.

### StarBase Database Analysis

starBase (http://starbase.sysu.edu.cn/) is an open-source database for investigating non-coding RNA interactions from CLIP-seq, degradome-seq and RNA-RNA interactome data (19, 20). starBase was introduced to perform expression correlation analysis for mRNA-miRNA and miRNA-lncRNA pairs in hepatocellular carcinoma. *R* < −0.1 and *P* < 0.05 were set as the criteria for identifying significant interactions. miRNA expression values in hepatocellular carcinoma were also determined using starBase. *P* < 0.05 was considered as statistically significant. Besides, starBase was employed to predict potential lncRNAs binding to miRNAs.

### Kaplan-Meier Plotter Analysis

Kaplan-Meier plotter database is capable to assess the effect of miRNAs and genes on survival in 21 cancer types, including hepatocellular carcinoma (21). The prognostic values of potential miRNAs in hepatocellular carcinoma was evaluated using Kaplan-Meier plotter (http://kmplot.com/analysis/) as we previously described (22, 23). In brief, each miRNA of interest was first entered into this database. According to median expression value, all cases were classified into a low expression group and a high expression group. Subsequently, Kaplan-Meier survival plots were generated, and statistical indices containing hazard ratio (HR), 95% confidence interval (CI), logrank *P*-value were automatically calculated and directly displayed on the webpage. Logrank *P* < 0.05 was considered as statistically significant.

### Cell Culture and Clinical Tissues

Human hepatocellular carcinoma cell lines hepG2 and LM3 and normal hepatic cell line HL7702 used in this study were kindly provided by the First Affiliated Hospital of Medical College, Zhejiang University (Hangzhou, China). All cell lines were cultured in Dulbecco's modified Eagle's medium (DMEM; Gibco, 12430047) supplemented with 10% fetal bovine serum (FBS; Biological Industries, 04-0101-1, Cromwell, CT, USA) under a humidified atmosphere of 5% CO_2_ at 37°C. Twenty-two clinical hepatocellular carcinoma tissues and matched adjacent normal hepatic tissues were collected from hepatocellular carcinoma patients who underwent surgery at the First Affiliated Hospital of Medical College, Zhejiang University (Hangzhou, China). All procedures performed in this study involving human participants were conducted in accordance with the ethical standards of the First Affiliated Hospital of Medical College, Zhejiang University. The written informed consent from every participant was obtained.

### RNA Extraction, Reverse Transcription PCR and Quantitative Real-Time PCR (qRT-PCR)

RNA extraction, reverse transcription PCR and quantitative real-time fluorescence PCR were conducted as previously described (24, 25). Total RNA was first extracted from cells and clinical samples using RNAiso plus Reagent (TaKaRa, Kusatsu, Japan), after which total RNA was reversely transcribed into complementary DNA (cDNA) by the PrimeScript^TM^ RT Reagent Kit (TaKaRa, RR037A). Then, qRT-PCR was performed using SYBR Premix Ex Taq (TaKaRa, RR420A) in a Roche LightCycle480 II Real-Time PCR Detection System. GAPDH was employed as the internal control for gene expression analysis. Gene expression was normalized to GAPDH and calculated through the comparative threshold method of 2^−ΔΔ*CT*^. All primers used in this study were listed Table S1.

### Cell Transfection

20 × 10^4^ of hepG2 and LM3 cells were seeded into 6-well plates and cultured for 12 h. Then, miRNA mimics and negative controls were transfected into these cells using Opti-MEM and Lipofectamine 3000 reagents (Invitrogen, Shanghai, China) according to the manufacturer's instruction. At 12 h post-transfection, the medium was replaced with fresh DMEM. The miRNA mimics and negative controls were synthesized and purchased from RiboBio Co. Ltd. (Guangzhou, China).

### Statistical Analysis

Most of the statistical analyses were done by the bioinformatic online tools as mentioned above. *P*-values from GEPIA expression analysis, logrank *P*-values from GEPIA and Kaplan-Meier plotter survival analysis were corrected by false discovery rate and other reported *P*-values by online tools were not adjusted for false discovery rate correction. The statistical analyses of experimental data were conducted by GraphPad Prism software (version 7.0.3). Experiments were performed in triplicates and shown as mean ± standard deviation (SD) from at least three independent times.

Student's *t*-test (two tailed) were employed to do comparisons between two groups. ROC curve was utilized to assess diagnostic effect. Logrank *P* < 0.05 or *P* < 0.05 was considered as statistically significant.

## Results

### CELSR3, GPSM2, and CHEK1 Were Identified as Three Novel Prognosis-Associated Genes in Hepatocellular Carcinoma

**Figure 1:**
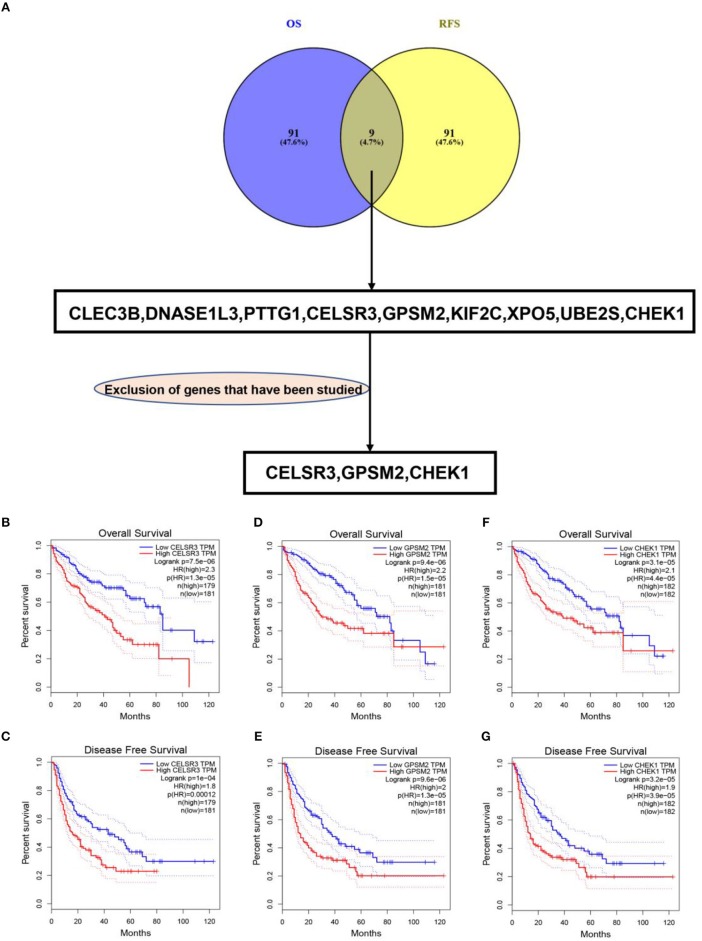
Identification of novel genes significantly associated with prognosis of hepatocellular carcinoma. **(A)** Identification of CELSR3, GPSM2, and CHEK1 as three novel genes linked to prognosis of hepatocellular carcinoma by intersecting the genes significantly associated with overall survival (OS) and disease-free survival (RFS) and excluding those genes previously studied; high expression of CELSR3 indicated poor OS **(B)** and RFS **(C)** of hepatocellular carcinoma; high expression of GPSM2 indicated poor OS **(D)** and RFS **(E)** of hepatocellular carcinoma; high expression of CHEK1 indicated poor OS **(F)** and RFS **(G)** of hepatocellular carcinoma. Logrank *P* < 0.05 was considered as statistically significant.

To obtain the genes most associated with patient survival in hepatocellular carcinoma, GEPIA database was first utilized. In this study, two indices regarding to patients' outcome, overall survival (OS) and disease-free survival (RFS), were included. The top 100 OS-associated genes and the top 100 RFS-associated genes were identified as listed in Tables 1, 2, respectively. By intersecting OS-associated genes and RFS-associated genes, 9 genes (CLEC3B, DNASE1L3, PTTG1, CELSR3, GPSM2, KIF2C, XPO5, UBE2S, and CHEK1) were defined as candidate genes, which were commonly appeared in OS-associated gene set and RFS-associated gene set (Figure 1A). After reviewing the published literatures and previous studies, we found that 6 of 9 genes [CLEC3B (26), DNASE1L3 (27), PTTG1 (28, 29), KIF2C (30), XPO5 (31, 32), and UBE2S (33, 34)] have been demonstrated to act as promising prognostic biomarkers for hepatocellular carcinoma. The rest three genes (CELSR3, GPSM2, and CHEK1) have not been studied for their prognostic values in hepatocellular carcinoma so far. Therefore, CELSR3, GPSM2 and CHEK1 were considered as three novel potential prognostic biomarkers for hepatocellular carcinoma. The prognostic values (OS and RFS) of CELSR3, GPSM2, and CHEK1 were presented in Figures 1B–G. The results suggested that high expression of CELSR3, GPSM2, or CHEK1 indicated poor prognosis in patients with hepatocellular carcinoma.

**Table 1:** The genes most associated with overall survival (OS) of patients with hepatocellular carcinoma determined by GEPIA database.

**Gene symbol**	**Gene ID**	**Logrank *P*-value**
HILPDA	ENSG00000135245.9	0.0000000076
CCDC58	ENSG00000160124.9	0.0000000558
B3GAT3	ENSG00000149541.9	0.00000023
CLEC3B	ENSG00000163815.5	0.000000243
CTB-147N14.6	ENSG00000275719.1	0.000000255
SOCS2	ENSG00000120833.13	0.000000917
LPCAT1	ENSG00000153395.9	0.00000125
PES1	ENSG00000100029.17	0.00000179
DNASE1L3	ENSG00000163687.13	0.00000187
AHSA1	ENSG00000100591.7	0.00000192
RP11-286H15.1	ENSG00000272789.1	0.00000225
SAC3D1	ENSG00000168061.13	0.00000234
SEC61G	ENSG00000132432.13	0.00000293
RP11-295D4.1	ENSG00000262712.1	0.00000297
RHPN1-AS1	ENSG00000254389.3	0.00000365
MTCH1	ENSG00000137409.18	0.00000369
PTTG1	ENSG00000164611.12	0.00000379
SAMD13	ENSG00000203943.8	0.00000388
CDC20	ENSG00000117399.13	0.000004
RPUSD3	ENSG00000156990.14	0.00000424
SLC41A3	ENSG00000114544.15	0.00000436
STEAP1B	ENSG00000105889.14	0.00000518
CCDC137	ENSG00000185298.12	0.00000563
MED19	ENSG00000156603.14	0.00000611
SNRPEP2	ENSG00000256968.1	0.00000686
LINC01134	ENSG00000236423.5	0.00000698
NRAV	ENSG00000248008.2	0.00000704
TMEM185B	ENSG00000226479.3	0.00000708
HM13	ENSG00000101294.16	0.00000714
NAP1L1P1	ENSG00000254759.1	0.00000758
CELSR3	ENSG00000008300.14	0.00000768
GNL2	ENSG00000134697.12	0.00000847
EIF2S2	ENSG00000125977.6	0.00000941
GPSM2	ENSG00000121957.12	0.00000964
TIMM23	ENSG00000265354.3	0.00000985
PPM1G	ENSG00000115241.10	0.00000989
BTNL9	ENSG00000165810.16	0.00001
SLC29A3	ENSG00000198246.7	0.0000101
KIF2C	ENSG00000142945.12	0.0000108
MPV17	ENSG00000115204.14	0.000011
SCML2	ENSG00000102098.17	0.0000122
TMEM251	ENSG00000153485.5	0.0000122
MUTYH	ENSG00000132781.17	0.000013
EIF2B5	ENSG00000145191.11	0.0000134
LRRC41	ENSG00000132128.16	0.0000141
ATP1B3	ENSG00000069849.10	0.0000142
UQCRH	ENSG00000173660.11	0.0000143
RAB42	ENSG00000188060.6	0.0000143
DTYMK	ENSG00000168393.12	0.0000144
PIGU	ENSG00000101464.10	0.0000144
FAM189B	ENSG00000160767.20	0.0000145
ZNF576	ENSG00000124444.15	0.0000151
RRP12	ENSG00000052749.13	0.0000154
TAF3	ENSG00000165632.7	0.0000163
GARS	ENSG00000106105.13	0.0000169
XPO5	ENSG00000124571.17	0.0000169
ERGIC3	ENSG00000125991.18	0.0000172
CLTA	ENSG00000122705.16	0.0000177
SLC16A3	ENSG00000141526.14	0.000018
KIFC1	ENSG00000237649.7	0.0000198
EIF5B	ENSG00000158417.10	0.0000215
CMB9-22P13.1	ENSG00000173727.11	0.0000221
TRPC4AP	ENSG00000100991.11	0.0000224
GAPDH	ENSG00000111640.14	0.0000227
CCT3	ENSG00000163468.14	0.0000232
BRK1	ENSG00000254999.3	0.0000232
CDC42EP2	ENSG00000149798.4	0.0000234
TMEM106C	ENSG00000134291.11	0.0000257
POLR2L	ENSG00000177700.5	0.0000264
UBE2S	ENSG00000108106.13	0.0000278
COMMD3	ENSG00000148444.15	0.0000279
PPP1R14B	ENSG00000173457.10	0.0000292
MRPL11	ENSG00000174547.13	0.0000294
SLC11A1	ENSG00000018280.16	0.00003
CHEK1	ENSG00000149554.12	0.0000303
SRD5A3	ENSG00000128039.10	0.0000311
UCK2	ENSG00000143179.12	0.0000317
MKLN1-AS	ENSG00000236753.5	0.0000335
RP5-864K19.4	ENSG00000228436.2	0.0000343
KRBA1	ENSG00000133619.17	0.0000345
TMEM147	ENSG00000105677.11	0.0000355
CTC-297N7.9	ENSG00000264016.2	0.0000357
HCFC1	ENSG00000172534.13	0.0000363
ILKAP	ENSG00000132323.8	0.0000364
AGTRAP	ENSG00000177674.15	0.0000364
MAPKAPK5-AS1	ENSG00000234608.7	0.000037
NKX3-2	ENSG00000109705.7	0.0000375
UPF3B	ENSG00000125351.10	0.0000377
RRP8	ENSG00000132275.10	0.0000416
ABCC5	ENSG00000114770.16	0.0000422
C4orf47	ENSG00000205129.8	0.0000423
CDKN2C	ENSG00000123080.10	0.0000426
CCT4	ENSG00000115484.14	0.0000437
NUP37	ENSG00000075188.8	0.0000442
RAB24	ENSG00000169228.13	0.0000445
MED10	ENSG00000133398.3	0.0000456
MIR210HG	ENSG00000247095.2	0.0000458
SLC1A5	ENSG00000105281.12	0.0000467
SMS	ENSG00000102172.15	0.000047
AP001469.9	ENSG00000239415.1	0.000047

**Table 2:** The genes most associated with disease-free survival (RFS) of patients with hepatocellular carcinoma determined by GEPIA database.

**Gene symbol**	**Gene ID**	**Logrank *P*-value**
XPO5	ENSG00000124571.17	0.000000141
PCNT	ENSG00000160299.16	0.000000524
RP11-218F10.3	ENSG00000273449.1	0.00000195
CCNB1	ENSG00000134057.14	0.00000219
AC010761.8	ENSG00000264577.1	0.00000257
BRD8	ENSG00000112983.17	0.00000315
RHOT2	ENSG00000140983.13	0.00000329
MYO19	ENSG00000278259.4	0.00000341
CDC25C	ENSG00000158402.18	0.00000642
GPSM2	ENSG00000121957.12	0.00000688
HIST1H1B	ENSG00000184357.4	0.00000743
STX1A	ENSG00000106089.11	0.00000879
TRIM45	ENSG00000134253.9	0.00000912
NUP85	ENSG00000125450.10	0.0000102
CENPK	ENSG00000123219.12	0.0000112
LRP11	ENSG00000120256.9	0.0000117
XCR1	ENSG00000173578.7	0.0000138
CLEC3B	ENSG00000163815.5	0.0000145
RAD51C	ENSG00000108384.14	0.0000153
BRCA1	ENSG00000012048.19	0.0000154
PRR3	ENSG00000204576.11	0.0000156
CAD	ENSG00000084774.13	0.0000159
MCM6	ENSG00000076003.4	0.0000164
RPL14P3	ENSG00000241923.2	0.0000168
PSMC3IP	ENSG00000131470.14	0.0000174
ADH4	ENSG00000198099.8	0.0000177
PTTG1	ENSG00000164611.12	0.0000186
MED7	ENSG00000155868.7	0.0000199
SRP68	ENSG00000167881.14	0.0000207
SNHG20	ENSG00000234912.9	0.0000219
PARPBP	ENSG00000185480.11	0.0000221
CHAF1B	ENSG00000159259.7	0.0000224
RP11-197P3.5	ENSG00000229587.2	0.0000232
CHEK1	ENSG00000149554.12	0.0000241
KPNA2	ENSG00000182481.8	0.0000254
E2F8	ENSG00000129173.12	0.0000258
C2orf66	ENSG00000187944.2	0.0000266
EFTUD2	ENSG00000108883.12	0.0000266
SCRT1	ENSG00000261678.2	0.0000272
RP11-127B20.3	ENSG00000272677.1	0.0000283
WI2-89031B12.1	ENSG00000261773.1	0.0000302
DHX57	ENSG00000163214.20	0.0000319
MKI67	ENSG00000148773.12	0.0000337
LMNB1	ENSG00000113368.11	0.0000338
RP11-709D24.8	ENSG00000278434.1	0.0000349
UBE2S	ENSG00000108106.13	0.0000353
MCM10	ENSG00000065328.16	0.0000366
RPL39P3	ENSG00000235174.1	0.0000422
NRM	ENSG00000137404.14	0.0000424
ZNF131	ENSG00000172262.11	0.0000428
LCN6	ENSG00000267206.5	0.0000435
RAD54L	ENSG00000085999.11	0.0000444
SGOL1	ENSG00000129810.14	0.0000448
KIF2C	ENSG00000142945.12	0.0000449
OLFM1	ENSG00000130558.18	0.0000458
FAM216A	ENSG00000204856.11	0.000047
RP5-967N21.11	ENSG00000275632.1	0.0000475
MCM3	ENSG00000112118.17	0.0000479
WBP2NL	ENSG00000183066.14	0.0000484
WDR4	ENSG00000160193.11	0.0000501
SLC25A19	ENSG00000125454.11	0.0000509
NUP205	ENSG00000155561.14	0.0000519
LINC01268	ENSG00000227502.2	0.0000522
STAG3	ENSG00000066923.17	0.0000534
RNFT2	ENSG00000135119.14	0.0000535
C5orf45	ENSG00000161010.14	0.0000538
RP13-516M14.1	ENSG00000260563.3	0.0000542
STMN1	ENSG00000117632.20	0.0000549
RBM28	ENSG00000106344.8	0.0000588
ZWINT	ENSG00000122952.16	0.0000598
EME1	ENSG00000154920.14	0.00006
PHF19	ENSG00000119403.13	0.0000638
C12orf43	ENSG00000157895.11	0.0000643
DNASE1L3	ENSG00000163687.13	0.000066
CENPH	ENSG00000153044.9	0.0000663
AP000695.4	ENSG00000233818.1	0.0000701
UTP18	ENSG00000011260.13	0.0000716
CTD-2349P21.9	ENSG00000266490.1	0.0000729
NASP	ENSG00000132780.16	0.0000758
POLR3F	ENSG00000132664.11	0.0000763
AGAP10P	ENSG00000230869.1	0.0000764
EZH2	ENSG00000106462.10	0.0000766
DDAH2	ENSG00000213722.8	0.0000766
HEATR5A	ENSG00000129493.14	0.0000786
RP11-225B17.2	ENSG00000273014.1	0.0000786
RP5-1074L1.4	ENSG00000273373.1	0.0000794
TBRG4	ENSG00000136270.13	0.0000799
OMG	ENSG00000126861.4	0.00008
AXIN1	ENSG00000103126.14	0.0000801
NR2C1	ENSG00000120798.16	0.0000802
RP11-932O9.10	ENSG00000269974.1	0.0000815
SPEF1	ENSG00000101222.12	0.0000867
BFSP1	ENSG00000125864.11	0.0000876
ATRIP	ENSG00000164053.17	0.0000879
SPATA2	ENSG00000158480.10	0.000091
CELSR3	ENSG00000008300.14	0.0000925
CCZ1B	ENSG00000146574.15	0.0000933
JCHAIN	ENSG00000132465.10	0.0000936
UTP6	ENSG00000108651.9	0.0000941
RP4-620E11.8	ENSG00000273951.1	0.0000973

### CELSR3, GPSM2, and CHEK1 Were Upregulated in Hepatocellular Carcinoma

**Figure 2:**
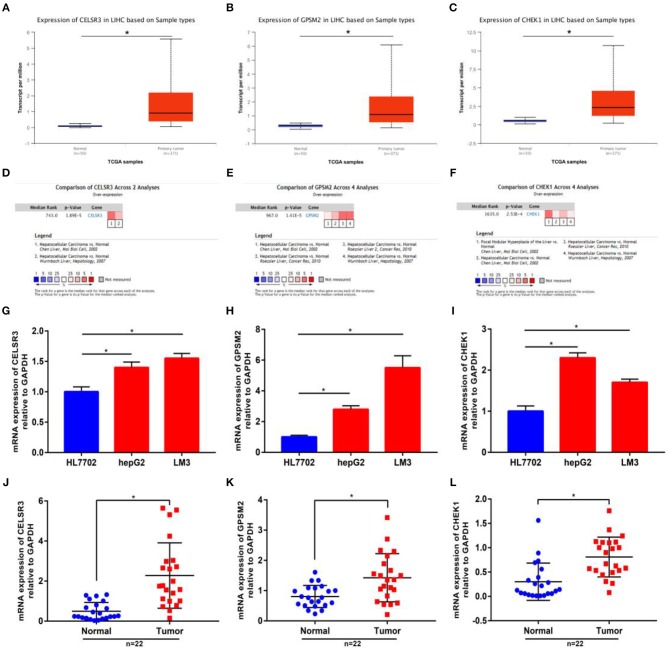
The mRNA expression levels of CELSR3, GPSM2, and CHEK1 in hepatocellular carcinoma. mRNA expression of CELSR3 **(A)**, GPSM2 **(B)**, and CHEK1 **(C)** were significantly increased in tumor tissues compared to normal controls determined by UALCAN database; mRNA expression of CELSR3 **(D)**, GPSM2 **(E)**, and CHEK1 **(F)** were significantly increased in tumor tissues compared to normal controls determined by Oncomine database. CELSR3 **(G)**, GPSM2 **(H)**, and CHEK1 **(I)** were markedly upregulated in hepG2 and LM3 when compared with HL7702; mRNA expression of CELSR3 **(J)**, GPSM2 **(K)**, and CHEK1 **(L)** were obviously higher in tumor tissues than that in adjacent normal tissues; normalization was done relative to GAPDH. *P < 0.05.

Next, we intended to determine expression levels of three novel prognosis-associated genes in hepatocellular carcinoma by bioinformatic analysis and experimental validation. Firstly, we detected their expression in TCGA hepatocellular carcinoma tissues and normal tissues using UALCAN database. CELSR3, GPSM2, and CHEK1 were significantly upregulated in tumor samples compared with normal samples as shown in Figures 2A–C, respectively. Next, Oncomine database was further introduced to analyze CELSR3, GPSM2, and CHEK1 expression in hepatocellular carcinoma. The datasets met the thresholds of *P* < 0.05 and |fold change| > 1.5 were included for conducting meta-analysis. As presented in Figures 2D–F, CELSR3, GPSM2, and CHEK1 expression were markedly higher in hepatocellular carcinoma than that in normal controls. Protein expression levels of CELSR3, GPSM2, and CHEK1 were evaluated using HumanProteinAtlas database. Figures S1A,B suggested that both CELSR3 and GPSM2 protein expression levels were increased in tumor tissue compared to normal tissue. However, CHEK1 protein value was not included in HumanProteinAtlas database. Subsequently, using qRT-PCR, we found that expression levels of CELSR3 (Figure 2G), GPSM2 (Figure 2H), and CHEK1 (Figure 2I) were obviously increased in two hepatocellular carcinoma cell lines (hepG2 and LM3) when compared with normal hepatic cell line (HL7702). Moreover, compared to normal hepatic tissues, CELSR3, GPSM2, and CHEK1 expression levels were significantly upregulated in collected hepatocellular carcinoma clinical tissues as shown in Figures 2J–L, respectively. All these findings demonstrate that CELSR3, GPSM2, and CHEK1 were upregulated in hepatocellular carcinoma and linked to prognosis of patients with hepatocellular carcinoma.

### Prediction and Validation of Potential miRNAs Binding to CELSR3, GPSM2, and CHEK1

**Figure 3:**
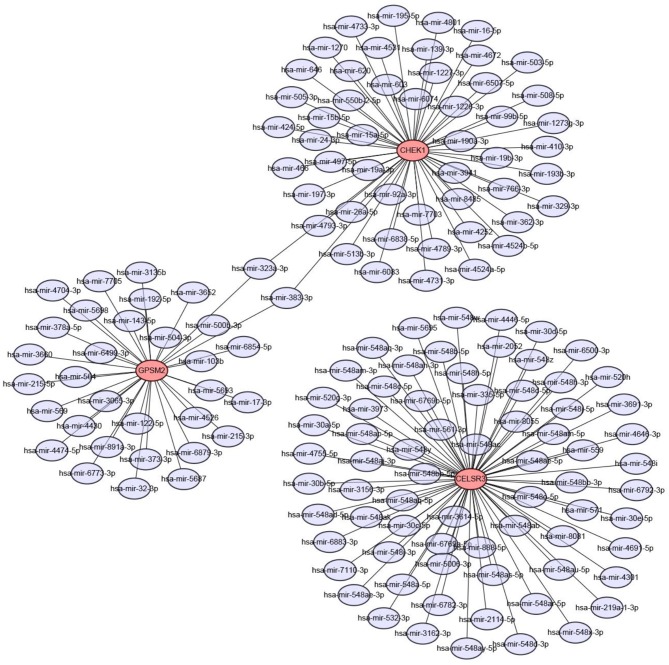
Construction of CELSR3/GPSM2/CHEK1-miRNA network by miRNet database and Cytoscape software.

**Figure 4:**
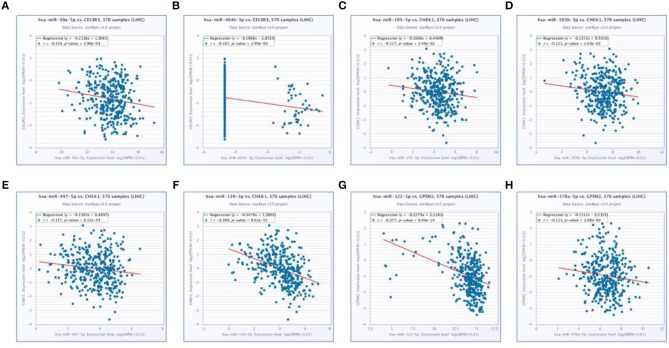
Significantly correlated mRNA-miRNA pairs determined by starBase database. **(A)** Expression of hsa-mir-30a-5p was negatively associated with CELSR3 expression; **(B)** expression of hsa-mir-4646-3p was negatively associated with CELSR3 expression; **(C)** expression of hsa-mir-195-5p was negatively associated with CHEK1 expression; **(D)** expression of hsa-mir-193b-3p was negatively associated with CHEK1 expression; **(E)** expression of hsa-mir-497-5p was negatively associated with CHEK1 expression; **(F)** expression of hsa-mir-139-3p was negatively associated with CHEK1 expression; **(G)** expression of hsa-mir-122-5p was negatively associated with GPSM2 expression; **(H)** expression of hsa-mir-378a-5p was negatively associated with GPSM2 expression.

**Figure 5:**
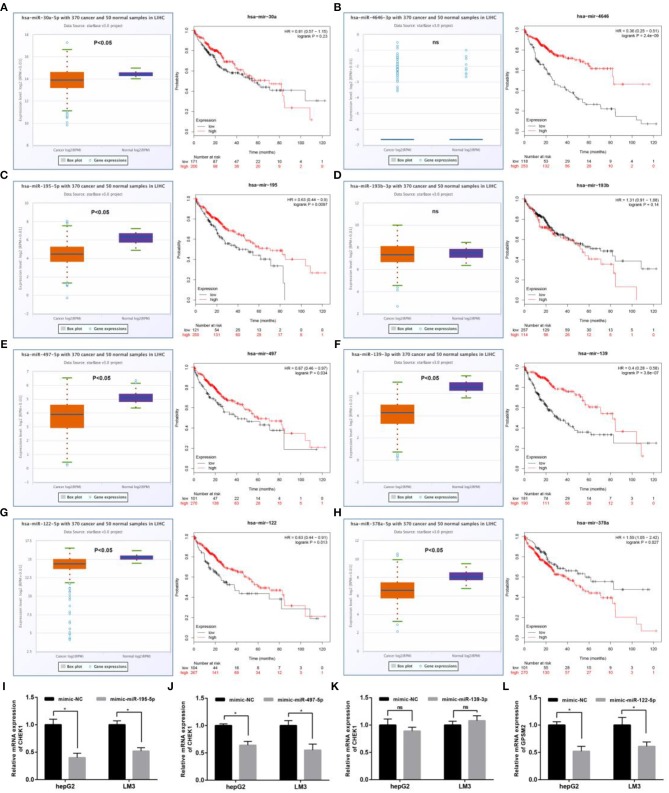
Identification of the most potential miRNAs associated with prognosis of hepatocellular carcinoma. Expression and prognostic value of hsa-mir-30a-5p **(A)**, hsa-mir-4646-3p **(B)**, hsa-mir-195-5p **(C)**, hsa-mir-193b-3p **(D)**, hsa-mir-497-5p **(E)**, hsa-mir-139-3p **(F)**, hsa-mir-122-5p **(G)**, and hsa-mir-378a-5p **(H)** in hepatocellular carcinoma; **(I)** after overexpression of hsa-mir-195-5p, mRNA expression of CHEK1 was significantly decreased in hepG2 and LM3; **(J)** after overexpression of hsa-mir-497-5p, mRNA expression of CHEK1 was significantly decreased in hepG2 and LM3; **(K)** after overexpression of hsa-mir-139-3p, no significant downregulation of CHEK1 mRNA expression was observed in hepG2 and LM3; **(L)** after overexpression of hsa-mir-122-5p, mRNA expression of GPSM2 was significantly decreased in hepG2 and LM3. *P < 0.05.

Next, we predicted upstream regulatory miRNAs of CELSR3, GPSM2, and CHEK1 through a comprehensive miRNA study-associated database, miRNet. A total of 156 mRNA-miRNA pairs, including 71 CELSR3-miRNA pairs, 52 GPSM2-miRNA pairs and 33 CHEK1-miRNA pairs, were acquired. For better visualization, mRNA-miRNA interactive network was constructed using Cytoscape software as presented in Figure 3. According to the classic action mechanism of miRNA in negative regulation of gene expression, there should be inverse expression relationship between the predicted mRNA-miRNA interactions. Thus, we employed starBase database to perform expression correlation analysis for these mRNA-miRNA interactions in hepatocellular carcinoma. The analytic results were shown in Table S2. Those mRNA-miRNA pairs with *R* < −0.1 and *P* < 0.05 were considered as significant interactions. Among the 156 interactions, only 8 mRNA-miRNA pairs, including CELSR3-hsa-mir-30a-5p, CELSR3-hsa-mir-4646-3p, CHEK1-hsa-mir-195-5p, CHEK1-hsa-mir-193b-3p, CHEK1-hsa-mir-497-5p, CHEK1-hsa-mir-139-3p, GPSM2-hsa-mir-122-5p, and GPSM2-hsa-mir-378a-5p, were identified as significant interactions (Figure 4). Theoretically, miRNAs that potentially bind to oncogenic CELSR3, GPSM2, and CHEK1 should be downregulated in hepatocellular carcinoma and display favorable prognostic roles. The expression levels of these potential miRNAs and their prognostic roles in hepatocellular carcinoma were determined using starBase database and Kaplan-Meier plotter database, respectively. By combination of expression analysis and survival analysis, hsa-mir-195-5p, hsa-mir-497-5p, hsa-mir-139-3p, and hsa-mir-122-5p were the potential miRNAs in hepatocellular carcinoma (Figures 5A–H). Subsequently, we further determined expression change of target gene (CHEK1 or GPSM2) after overexpression of hsa-mir-195-5p, hsa-mir-497-5p, hsa-mir-139-3p, or hsa-mir-122-5p in hepG2 and LM3 cell lines. Figures 5I,J showed a significant reduction of CHEK1 expression after overexpression of hsa-mir-195-5p and hsa-mir-497-5p; Figure 5K revealed that upregulation of hsa-mir-139-3p did not influence CHEK1 expression; Figure 5L demonstrated that GPSM2 expression was significantly downregulated after overexpression of hsa-mir-122-5p. All these results together indicate that CHEK1-hsa-mir-195-5p/hsa-mir-497-5p and GPSM2-hsa-mir-122-5p may be key pathways in mediating progression of hepatocellular carcinoma and that link to patients' prognosis.

### Prediction and Validation of Key lncRNAs Binding to Potential miRNAs

**Figure 6:**
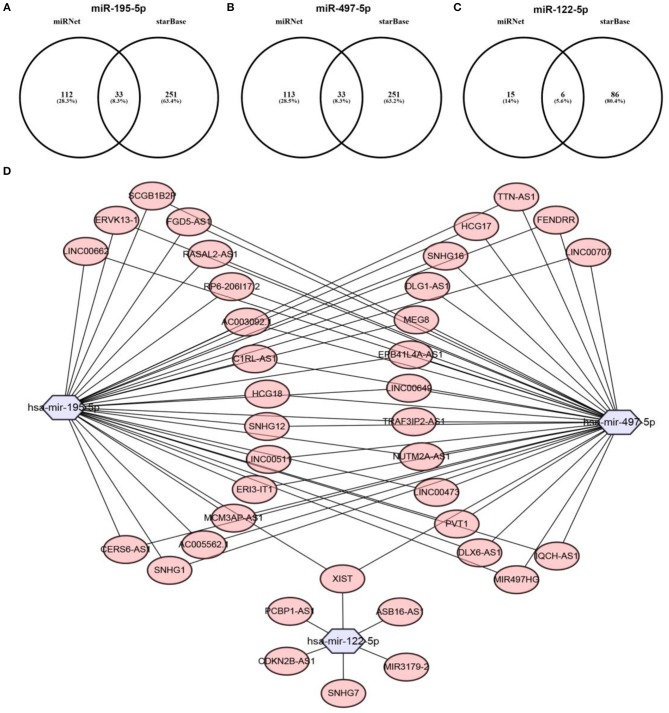
Prediction of upstream lncRNAs potentially binding to hsa-mir-195-5p, hsa-mir-497-5p, and hsa-mir-122-5p. **(A)** Potential lncRNAs of hsa-mir-195-5p predicted by miRNet and starBase databases; **(B)** potential lncRNAs of hsa-mir-497-5p predicted by miRNet and starBase databases; **(C)** potential lncRNAs of hsa-mir-122-5p predicted by miRNet and starBase databases; **(D)** establishment of miRNA-lncRNA network using Cytoscape software.

**Figure 7:**
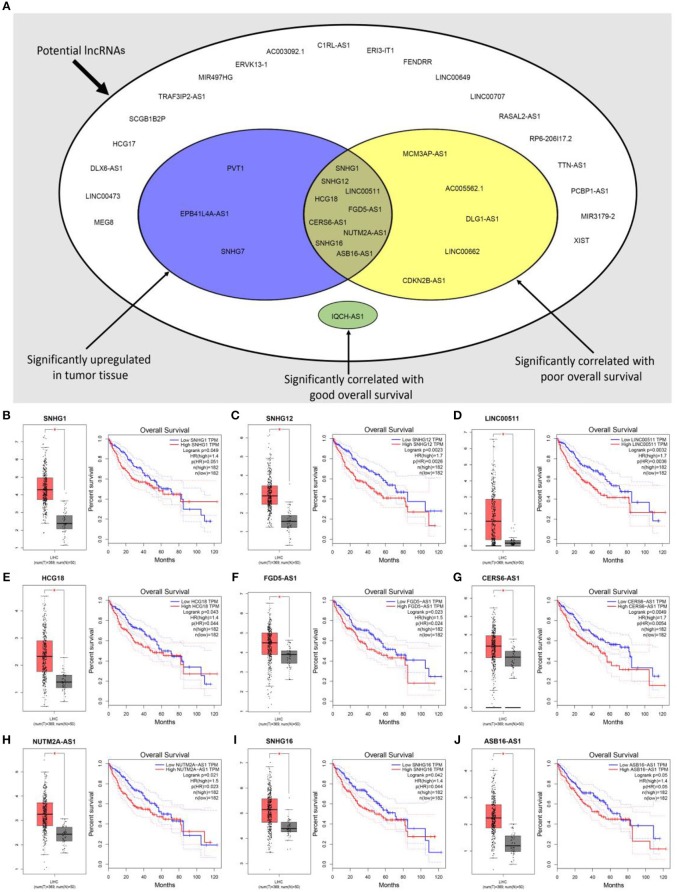
Expression analysis and survival analysis of potential lncRNAs in hepatocellular carcinoma. **(A)** SNHG1, SNHG12, LINC00511, HCG18, FGD5-AS1, CERS6-AS1, NUTM2A-AS1, SNHG16, and ASB16-AS1 were identified as key lncRNAs in hepatocellular carcinoma by combination of their expression levels and prognostic values; **(B)** SNHG1 was significantly upregulated in tumor tissues and linked to poor prognosis of hepatocellular carcinoma; **(C)** SNHG12 was significantly upregulated in tumor tissues and linked to poor prognosis of hepatocellular carcinoma; **(D)** LINC00511 was significantly upregulated in tumor tissues and linked to poor prognosis of hepatocellular carcinoma; **(E)** HCG18 was significantly upregulated in tumor tissues and linked to poor prognosis of hepatocellular carcinoma; **(F)** FGD5-AS1 was significantly upregulated in tumor tissues and linked to poor prognosis of hepatocellular carcinoma; **(G)** CERS6-AS1 was significantly upregulated in tumor tissues and linked to poor prognosis of hepatocellular carcinoma; **(H)** NUTM2A-AS1 was significantly upregulated in tumor tissues and linked to poor prognosis of hepatocellular carcinoma; **(I)** SNHG16 was significantly upregulated in tumor tissues and linked to poor prognosis of hepatocellular carcinoma; **(J)** ASB16-AS1 was significantly upregulated in tumor tissues and linked to poor prognosis of hepatocellular carcinoma. *P < 0.05.

Previous studies have suggested that lncRNAs can bind to miRNA, and mediate regulation of target gene expression and play biological roles (35, 36). Thus, two databases, miRNet and starBase, were used to predict potential lncRNAs that may bind to hsa-mir-195-5p, hsa-mir-497-5p and hsa-mir-122-5p. One hundred and forty five and Two hundred and eighty four lncRNAs were predicted to target hsa-mir-195-5p by miRNet and starBase, respectively; 146 and 284 lncRNAs were predicted to target hsa-mir-497-5p by miRNet and starBase, respectively; 21 and 92 lncRNAs were predicted to target hsa-mir-122-5p by miRNet and starBase, respectively (Data were not shown). As shown in Figures 6A–C, 33, 33, and 6 lncRNAs binding to hsa-mir-195-5p, hsa-mir-497-5p, and hsa-mir-122-5p were commonly appeared in both miRNet and starBase databases. These lncRNAs were selected for subsequent analysis. For better visualization, the miRNA-lncRNA regulatory network was established by Cytoscape software (Figure 6D). Based on ceRNA hypothesis, lncRNAs targeting to hsa-mir-195-5p, hsa-mir-497-5p, and hsa-mir-122-5p should be oncogenic lncRNAs in hepatocellular carcinoma. By combination of expression analysis and survival analysis (Figure 7A), we found that nine lncRNAs, including SNHG1 (Figure 7B), SNHG12 (Figure 7C), LINC00511 (Figure 7D), HCG18 (Figure 7E), FGD5-AS1 (Figure 7F), CERS6-AS1 (Figure 7G), NUTM2A-AS1 (Figure 7H), SNHG16 (Figure 7I), and ASB16-AS1 (Figure 7J), were significantly upregulated in hepatocellular carcinoma, and their upregulation linked to poor prognosis of patients with hepatocellular carcinoma. The current findings support that SNHG1, SNHG12, LINC00511, HCG18, FGD5-AS1, CERS6-AS1, NUTM2A-AS1, SNHG16, and ASB16-AS1, upregulated and linked to poor prognosis in hepatocellular carcinoma, might be the most potential lncRNAs that bind to previous identified miRNAs, hsa-mir-195-5p, hsa-mir-497-5p, and hsa-mir-122-5p.

### Prediction and Validation of Upstream miRNA-lncRNA Network of “Known” mRNA in Hepatocellular Carcinoma

**Figure 8:**
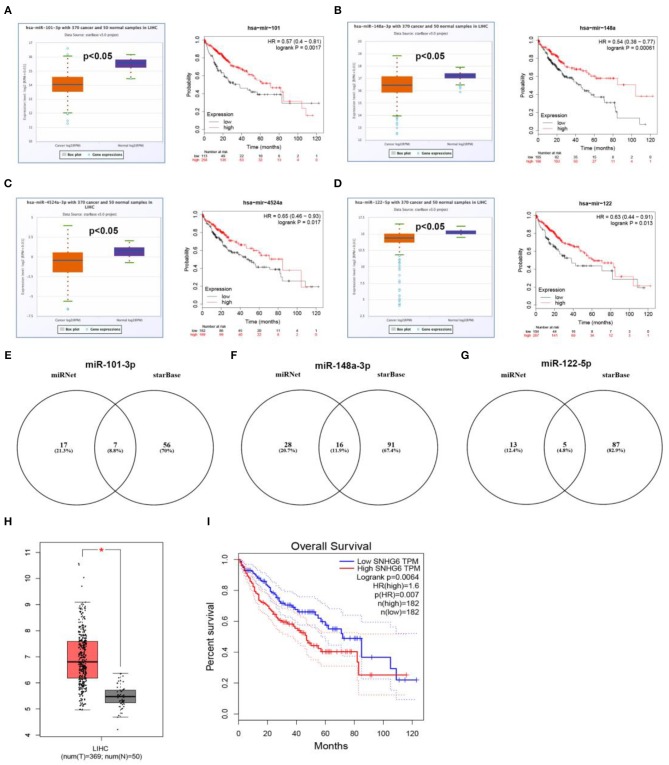
Prediction and validation of upstream miRNAs and lncRNAs of six “known” genes. Expression and prognostic value of hsa-mir-101-3p **(A)**, hsa-mir-148a-3p **(B)**, hsa-mir-4524a-3p **(C)**, hsa-mir-122-5p **(D)**. Potential lncRNAs of hsa-mir-101-3p **(E)**, hsa-mir-148a-3p **(F)**, hsa-mir-122-5p **(G)**, predicted by miRNet and starBase. **(H)** Expression of SNHG6 in hepatocellular carcinoma analyzed by GEPIA. **(I)** The prognostic value of SNHG6 in hepatocellular carcinoma assessed by GEPIA database.

Next, based on ceRNA mechanism, we further constructed a six “known” genes-miRNA-lncRNA network in hepatocellular carcinoma. Firstly, we predicted upstream miRNAs of CLEC3B, DNASE1L3, PTTG1, KIF2C, XPO5, and UBE2S using miRNet. As shown in Table S3, a total of 165 miRNA-mRNA pairs were predicted. Then, we performed expression correlation analysis for the 165 miRNA-mRNA pairs in hepatocellular carcinoma, and found that only 13 pairs presented significantly negative relationship about their expression (Table S3). Expression and survival analyses revealed that 4 miRNAs (hsa-miR-101-3p, hsa-miR-148a-3p, hsa-miR-4524a-3p, and hsa-miR-122-5p) were significantly downregulated in hepatocellular carcinoma and correlated with favorable prognosis (Figures 8A–D). Next, we predicted the upstream lncRNAs of the 4 miRNAs through miRNet and starBase databases. As presented in Figures 8E–G, 7, 16, and 5 lncRNAs were identified to potentially bind to hsa-miR-101-3p, hsa-miR-148a-3p, and hsa-miR-122-5p, respectively. No potential lncRNAs of hsa-miR-4524a-3p were found. Expression and survival analyses demonstrated that, among these predicted lncRNAs, SNHG1 (Figure 7B), HCG18 (Figure 7E), NUTM2A-AS1 (Figure 7H) and ASB16-AS1 (Figure 7J), and SNHG6 (Figures 8H,I) were markedly upregulated in hepatocellular carcinoma and linked to poor prognosis.

### Establishment of Key mRNA-miRNA-lncRNA Triple ceRNA Network in Hepatocellular Carcinoma

**Figure 9:**
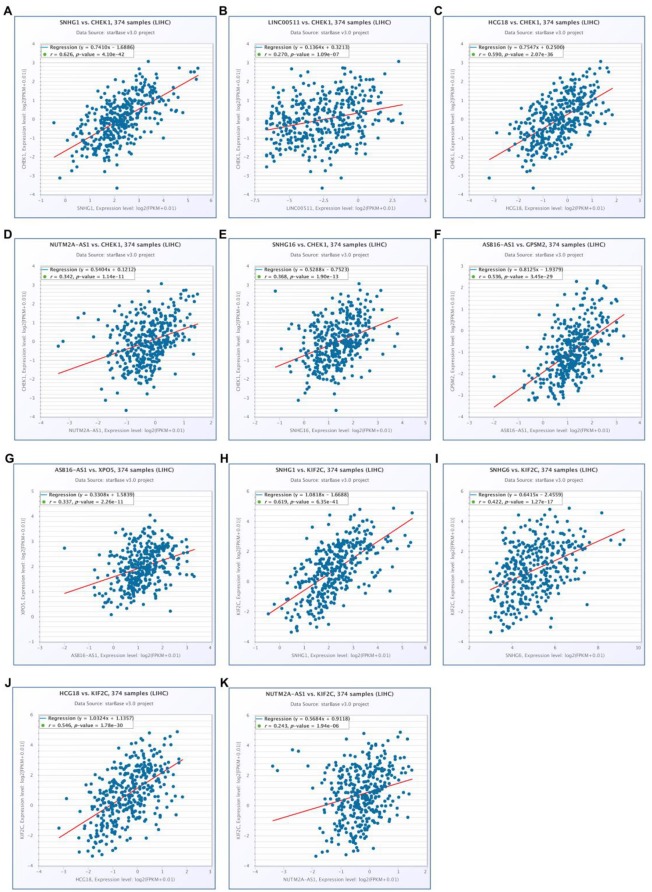
Correlation analysis of potential mRNA-lncRNA pairs in hepatocellular carcinoma determined by starBase. **(A)** SNHG1 expression was significantly positively correlated with CHEK1 expression in hepatocellular carcinoma; **(B)** LINC00511 expression was significantly positively correlated with CHEK1 expression in hepatocellular carcinoma; **(C)** HCG18 expression was significantly positively correlated with CHEK1 expression in hepatocellular carcinoma; **(D)** NUTM2A-AS1 expression was significantly positively correlated with CHEK1 expression in hepatocellular carcinoma; **(E)** SNHG16 expression was significantly positively correlated with CHEK1 expression in hepatocellular carcinoma; **(F)** ASB16-AS1 expression was significantly positively correlated with GPSM2 expression in hepatocellular carcinoma; **(G)** ASB16-AS1 expression was significantly positively correlated with XPO5 expression in hepatocellular carcinoma; **(H)** SNHG1 expression was significantly positively correlated with KIF2C expression in hepatocellular carcinoma; **(I)** SNHG6 expression was significantly positively correlated with KIF2C expression in hepatocellular carcinoma; **(J)** HCG18 expression was significantly positively correlated with KIF2C expression in hepatocellular carcinoma; **(K)** NUTM2A-AS1 expression was significantly positively correlated with KIF2C expression in hepatocellular carcinoma.

**Figure 10:**
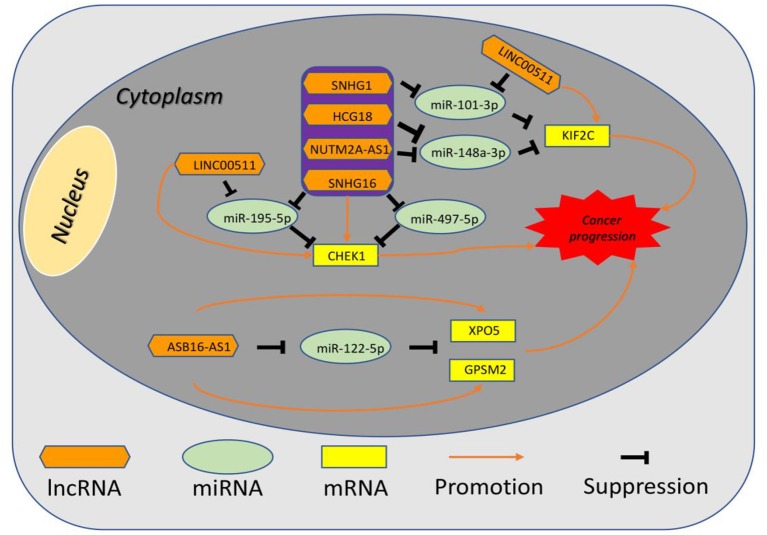
The established mRNA-miRNA-lncRNA competing endogenous RNA (ceRNA) triple network associated with progression and prognosis of hepatocellular carcinoma.

**Figure 11:**
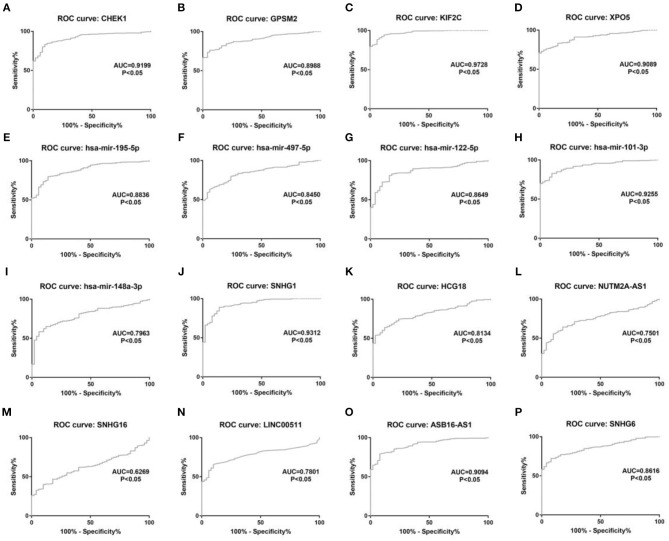
The diagnostic values of 16 potential molecules in hepatocellular carcinoma. **(A)** CHEK1 ROC analysis in hepatocellular carcinoma; **(B)** GPSM2 ROC analysis in hepatocellular carcinoma; **(C)** KIF2C ROC analysis in hepatocellular carcinoma; **(D)** XPO5 ROC analysis in hepatocellular carcinoma; **(E)** hsa-mir-195-5p ROC analysis in hepatocellular carcinoma; **(F)** hsa-mir-497-5p ROC analysis in hepatocellular carcinoma; **(G)** hsa-mir-122-5p ROC analysis in hepatocellular carcinoma; **(H)** hsa-mir-101-3p ROC analysis in hepatocellular carcinoma; **(I)** hsa-mir-148a-5p ROC analysis in hepatocellular carcinoma; **(J)** SNHG1 ROC analysis in hepatocellular carcinoma; **(K)** HCG18 ROC analysis in hepatocellular carcinoma; **(L)** NUTM2A-AS1 ROC analysis in hepatocellular carcinoma; **(M)** SNHG16 ROC analysis in hepatocellular carcinoma; **(N)** LINC00511 ROC analysis in hepatocellular carcinoma; **(O)** ASB16-AS1 ROC analysis in hepatocellular carcinoma. **(P)** SNHG6 ROC analysis in hepatocellular carcinoma. ROC, receiver operating characteristic. *P* < 0.05 was considered as statistically significant.

10 potential lncRNAs (SNHG1, SNHG12, LINC00511, HCG18, FGD5-AS1, CERS6-AS1, NUTM2A-AS1, SNHG16, ASB16-AS1, SNHG6) together with 5 potential miRNAs (hsa-miR-195-5p, hsa-miR-497-5p, hsa-miR-122-5p, hsa-miR-101-3p, and hsa-miR-148a-3p) made up a miRNA-lncRNA sub-network. After performing expression correlation analysis, we discovered that 14 of 21 interactions (hsa-miR-195-5p-SNHG1, hsa-miR-195-5p-LINC00511, hsa-miR-195-5p-HCG18, hsa-miR-195-5p-NUTM2A-AS1, hsa-miR-195-5p-SNHG16, hsa-miR-497-5p-SNHG1, hsa-miR-497-5p-HCG18, hsa-miR-497-5p-NUTM2A-AS1, hsa-miR-497-5p-SNHG16, hsa-miR-122-5p-ASB16-AS1, hsa-miR-101-3p-SNHG1, hsa-miR-101-3p-SNHG6, hsa-miR-148a-3p-HCG18, and hsa-miR-148a-3p-NUTM2A-AS1) possessed significant negative expression relationships (Table 3). The 7 lncRNAs (SNHG1, LINC00511, HCG18, NUTM2A-AS1, SNHG16, ASB16-AS1, and SNHG6) may potentially modulate CHEK1, GPSM2, XPO5, and KIF2C by competitively binding to shared miRNAs (hsa-miR-195-5p, hsa-miR-497-5p, hsa-miR-122-5p, hsa-miR-101-3p, and has-miR-148a-3p). According to ceRNA hypothesis, there should be positive association between mRNA expression and lncRNA expression. Two databases, starBase and GEPIA, were employed to analyze expression correlation of 11 mRNA-lncRNA pairs (CHEK1-SNHG1, CHEK1-LINC00511, CHEK1-HCG18, CHEK1-NUTM2A-AS1, CHEK1-SNHG16, and GPSM2-ASB16-AS1, XPO5-ASB16-AS1, KIF2C-SNHG1, KIF2C-SNHG6, KIF2C-HCG18, and KIF2C-NUTM2A-AS1). Notably, as presented in Figure 9, Figure S2, significant positive expression associations of all these mRNA-lncRNA pairs were observed in both starBase and miRNet databases. By integration of results from *in silico* analysis and experimental validation, we established a key mRNA-miRNA-lncRNA triple regulatory network associated with prognosis of hepatocellular carcinoma (Figure 10). Finally, we wanted to ascertain if these RNAs in the established mRNA-miRNA-lncRNA triple regulatory network possessed diagnostic roles in hepatocellular carcinoma. As shown in Figure 11, each RNA, including 4 mRNAs (CHEK1, GPSM2, KIF2C, XPO5), 5 miRNAs (hsa-miR-195-5p, hsa-miR-497-5p, hsa-miR-122-5p, hsa-miR-101-3p, hsa-miR-148a-3p) and 7 lncRNAs (SNHG1, HCG18, NUTM2A-AS1, SNHG16, LINC00511, ASB16-AS1, SNHG6), exhibited high diagnostic value for distinguishing hepatocellular carcinoma samples from normal samples.

**Table 3:** The correlation between potential miRNA-lncRNA pairs identified by starBase (The pairs conformed to the ceRNA hypothesis are marked with **Bold type**).

**miRNA**	**lncRNA**	***R***	***P*-value**
**hsa-mir-195-5p**	**SNHG1**	**−0.151**	**0.004**
hsa-mir-195-5p	SNHG12	**–**0.095	0.067
**hsa-mir-195-5p**	**LINC00511**	**−0.147**	**0.004**
**hsa-mir-195-5p**	**HCG18**	**−0.196**	**0.000**
hsa-mir-195-5p	FGD5-AS1	0.050	0.334
hsa-mir-195-5p	CERS6-AS1	**–**0.003	0.960
**hsa-mir-195-5p**	**NUTM2A-AS1**	**−0.210**	**0.000**
**hsa-mir-195-5p**	**SNHG16**	**−0.164**	**0.002**
**hsa-mir-497-5p**	**SNHG1**	**−0.139**	**0.008**
hsa-mir-497-5p	SNHG12	**–**0.050	0.342
hsa-mir-497-5p	LINC00511	**–**0.085	0.103
**hsa-mir-497-5p**	**HCG18**	**−0.259**	**0.000**
hsa-mir-497-5p	FGD5-AS1	**–**0.050	0.338
hsa-mir-497-5p	CERS6-AS1	**–**0.012	0.825
**hsa-mir-497-5p**	**NUTM2A-AS1**	**−0.202**	**0.000**
**hsa-mir-497-5p**	**SNHG16**	**−0.273**	**0.000**
**hsa-mir-122-5p**	**ASB16-AS1**	**−0.291**	**0.000**
**hsa-mir-101-3p**	**SNHG1**	**−0.357**	**0.000**
**hsa-mir-101-3p**	**SNHG6**	**−0.383**	**0.000**
**hsa-mir-148a-3p**	**HCG18**	**−0.259**	**0.000**
**hsa-mir-148a-3p**	**NUTM2A-AS1**	**−0.202**	**0.000**

## Discussion

Hepatocellular carcinoma is notorious for its poor prognosis and high aggressiveness. Elucidation of molecular mechanisms of hepatocellular carcinoma pathogenesis and identification of promising biomarkers for diagnosis and prognosis of hepatocellular carcinoma are critical for updating therapeutic approaches and improving patients' outcome. ceRNA regulatory network has been reported to participate in initiation and progression of human cancers. To our knowledge, a comprehensive ceRNA regulatory network based on the order model of mRNA-miRNA-lncRNA in hepatocellular carcinoma has not been constructed so far. Therefore, we tried to establish a prognosis/diagnosis-associated mRNA-miRNA-lncRNA ceRNA triple sub-network in hepatocellular carcinoma. By performing survival (OS and RFS) analysis using TCGA hepatocellular carcinoma data, nine genes including three novel genes (CELSR3, GPSM2 and CHEK1) and six “known” genes [CLEC3B (26), DNASE1L3 (27), PTTG1 (28, 29), KIF2C (30), XPO5 (31, 32) and UBE2S (33, 34)] were identified as prognosis-associated genes in hepatocellular carcinoma. The prognostic values of the three novel genes in hepatocellular carcinoma have not been investigated so far. However, they have been well-documented to function as key oncogenes and biomarkers in various types of cancer. For example, Pan et al. (37) reported that CELSR3 may be a promising prognostic gene in head and neck squamous cell carcinoma; He et al. (38) demonstrated that GPSM2 facilitated tumor growth and metastasis; CHEK1 downregulation suppressed by hsa-mir-195-5p hindered growth and metastasis of non-small cell lung cancer (39). *In silico* analysis and experimental validation together suggested that the three genes were significantly upregulated in hepatocellular carcinoma. All these findings indicate that high expression of CELSR3, GPSM2, or CHEK1 links to unfavorable prognosis of patients with hepatocellular carcinoma.

As mentioned above, mRNA can cross-talk with lncRNA by competitively binding to shared miRNAs (6). Therefore, potential miRNAs of CELSR3, GPSM2, and CHEK1 and lncRNAs that bind to potential miRNAs were successively predicted. 154 miRNAs of CELSR3, GPSM2, and CHEK1 were first identified using miRNet database. Based on the action mechanism of miRNA on mRNA and putative oncogenic roles of CELSR3, GPSM2, and CHEK1, potential miRNAs should be tumor suppressive miRNAs and should be negatively correlated with CELSR3, GPSM2, or CHEK1. Accordingly, we identified 8 potential pairs (CELSR3-hsa-mir-30a-5p, CELSR3-hsa-mir-4646-3p, CHEK1-hsa-mir-195-5p, CHEK1-hsa-mir-193b-3p, CHEK1-hsa-mir-497-5p, CHEK1-hsa-mir-139-3p, GPSM2-hsa-mir-122-5p, and GPSM2-hsa-mir-378a-5p) by conducting correlation analysis for these mRNA-miRNA interactions in hepatocellular carcinoma. After performing expression analysis, survival analysis and qRT-PCR, three of eight mRNA-miRNA pairs (CHEK1-hsa-mir-195-5p, CHEK1-hsa-mir-497-5p, and GPSM2-hsa-mir-122-5p) were selected for subsequent analysis. Liu et al. (39) suggested that CHEK1 was a direct target of hsa-mir-195-5p in non-small cell lung cancer; Xie et al. (40) confirmed that CHEK1 was inversely regulated by hsa-miR-497-5p in hepatocellular carcinoma. In spite of lacking of evidence that has-mir-122-5p directly binds to CHEK1, the inhibitory effect of hsa-mir-122-5p in multiple human cancers has been widely reported (41–43). Our current findings together with these reports suggest that CHEK1-hsa-mir-195-5p, CHEK1-hsa-mir-497-5p, and GPSM2-hsa-mir-122-5p may be key pathways in pathogenesis of hepatocellular carcinoma. Next, lncRNAs that bind to hsa-mir-195-5p, hsa-mir-497-5p, or hsa-mir-122-5p were predicted by miRNet and starBase databases. According to ceRNA hypothesis, potential lncRNAs of hsa-mir-195-5p, hsa-mir-497-5p, or hsa-mir-122-5p should act as oncogenic lncRNAs in hepatocellular carcinoma. Among all predicted lncRNAs, nine lncRNAs (SNHG1, SNHG12, LINC00511, HCG18, FGD5-AS1, CERS6-AS1, NUTM2A-AS1, SNHG16, and ASB16-AS1) were significantly upregulated in hepatocellular carcinoma and their upregulation linked to poor prognosis of patients with hepatocellular carcinoma. Most of them have been reported to function as oncogenic lncRNAs in hepatocellular carcinoma. For example, SNHG1 was found to promote liver cancer development through inhibiting p53 expression *via* binding to DNMT1 (44); Lan et al. (45) demonstrated that SNHG12 facilitated tumorigenesis and metastasis by targeting miR-199a/b-5p in hepatocellular carcinoma (45); LINC00511 contributed to cell proliferation and metastasis by modulating miR-424 in hepatocellular carcinoma (46); SNHG16 was also reported to enhance proliferation, migration and invasion of hepatocellular carcinoma (47–49). Besides, a similar mRNA-miRNA-lncRNA analysis for six “known” genes was performed.

By integrating these mRNA-miRNA and miRNA-lncRNA interactions, a potential mRNA-miRNA-lncRNA ceRNA triple sub-network associated with prognosis of hepatocellular carcinoma was constructed. Moreover, ROC curve analysis for these RNAs in the established network revealed that each of them possessed high diagnostic value for hepatocellular carcinoma, further suggesting the key roles of this mRNA-miRNA-lncRNA sub-network in hepatocellular carcinoma. Of course, more corresponding studies need to be launched in the future.

## Conclusion

In conclusion, using *in silico* analysis and experimental validation, we established a comprehensive mRNA-miRNA-lncRNA triple ceRNA network in hepatocellular carcinoma. Each RNA in this network possesses significant expression difference between hepatocellular carcinoma and normal control and has promising diagnostic and prognostic values for patients with hepatocellular carcinoma. However, these findings need to be further confirmed by more basic experiments and larger-scale clinical trials in the future.

## Data Availability Statement

The datasets analyzed in this study can be found in the GEPIA, UALCAN, and Oncomine databases.

## Ethics Statement

The studies involving human participants were reviewed and approved by the Ethical Committee of First Affiliated Hospital of Zhejiang University School of Medicine. The patients/participants provided their written informed consent to participate in this study.

## Author Contributions

JZ designed the study, wrote the manuscript and performed *in silico* analysis of the data. WL conducted experiments and revised the manuscript. JZ and WL have read and approved the final manuscript.

### Conflict of Interest

The authors declare that the research was conducted in the absence of any commercial or financial relationships that could be construed as a potential conflict of interest.
