# Supplementary material for: A Key mRNA-miRNA-lncRNA Competing Endogenous RNA Triple Sub-network Linked to Diagnosis and Prognosis of Hepatocellular Carcinoma
Source: Front Oncol. 2020 Mar 17;10:340. doi: 10.3389/fonc.2020.00340 (PMC7092636; doi:10.3389/fonc.2020.00340)
Supplement: Supplementary file 1 [file Table_1.DOCX]

**Table S1.** The sequences of primers used in this study.

| Gene symbol | Primer | Sequence |
| --- | --- | --- |
| CELSR3 | Forward primer | 5′ GGTCAGGGAGCCTATCTTCGT 3′ |
|  | Reverse primer | 5′ CTGGACGCCGTGTTCAATC 3′ |
| GPSM2 | Forward primer | 5′ TGCAAGGACTATTGGAGACCA 3′ |
|  | Reverse primer | 5′ TCTGGAAATATCTAGGTGTCGCT 3′ |
| CHEK1 | Forward primer | 5′ ATATGAAGCGTGCCGTAGACT 3′ |
|  | Reverse primer | 5′ TGCCTATGTCTGGCTCTATTCTG 3′ |
| GAPDH | Forward primer | 5’ CTGGGCTACACTGAGCACC 3’ |
|  | Reverse primer | 5’ AAGTGGTCGTTGAGGGCAATG 3’ |
