# Supplementary material for: A Key mRNA-miRNA-lncRNA Competing Endogenous RNA Triple Sub-network Linked to Diagnosis and Prognosis of Hepatocellular Carcinoma
Source: Front Oncol. 2020 Mar 17;10:340. doi: 10.3389/fonc.2020.00340 (PMC7092636; doi:10.3389/fonc.2020.00340)
Supplement: Supplementary file 2 [file Table_2.DOCX]

**Table S2.** The correlation between miRNA-mRNA pairs determined by starBase database.

| mRNA | miRNA | R | P-value |
| --- | --- | --- | --- |
| CELSR3 | hsa-mir-30a-5p | -0.1540 | 0.003 |
| CELSR3 | hsa-mir-30c-5p | 0.0060 | 0.914 |
| CELSR3 | hsa-mir-30d-5p | -0.0930 | 0.075 |
| CELSR3 | hsa-mir-30b-5p | -0.0560 | 0.285 |
| CELSR3 | hsa-mir-30e-5p | 0.0510 | 0.327 |
| CELSR3 | hsa-mir-335-5p | 0.0050 | 0.930 |
| CELSR3 | hsa-mir-520g-3p | 0.1430 | 0.006 |
| CELSR3 | hsa-mir-520h | 0.1360 | 0.009 |
| CELSR3 | hsa-mir-559 | 0.0260 | 0.620 |
| CELSR3 | hsa-mir-561-3p | 0.0870 | 0.095 |
| CELSR3 | hsa-mir-571 | -0.0750 | 0.149 |
| CELSR3 | hsa-mir-548d-3p | 0.0700 | 0.176 |
| CELSR3 | hsa-mir-219a-1-3p | 0.1530 | 0.003 |
| CELSR3 | hsa-mir-532-3p | -0.0200 | 0.696 |
| CELSR3 | hsa-mir-548b-5p | 0.1850 | 0.000 |
| CELSR3 | hsa-mir-548a-5p | 0.0650 | 0.212 |
| CELSR3 | hsa-mir-548c-5p | 0.0000 | 1.000 |
| CELSR3 | hsa-mir-548d-5p | -0.0680 | 0.189 |
| CELSR3 | hsa-mir-888-5p | 0.0100 | 0.849 |
| CELSR3 | hsa-mir-548j-5p | 0.0800 | 0.126 |
| CELSR3 | hsa-mir-548h-5p | 0.0000 | 1.000 |
| CELSR3 | hsa-mir-548i | 0.0000 | 1.000 |
| CELSR3 | hsa-mir-2052 | 0.0000 | 1.000 |
| CELSR3 | hsa-mir-2114-5p | -0.0720 | 0.165 |
| CELSR3 | hsa-mir-548w | 0.0600 | 0.248 |
| CELSR3 | hsa-mir-548x-3p | 0.2190 | 0.000 |
| CELSR3 | hsa-mir-4301 | 0.0000 | 1.000 |
| CELSR3 | hsa-mir-3614-5p | -0.0740 | 0.154 |
| CELSR3 | hsa-mir-548y | 0.2760 | 0.000 |
| CELSR3 | hsa-mir-548z | 0.0000 | 1.000 |
| CELSR3 | hsa-mir-548ab | 0.0340 | 0.513 |
| CELSR3 | hsa-mir-548ac | -0.0840 | 0.108 |
| CELSR3 | hsa-mir-548ae-3p | 0.0000 | 1.000 |
| CELSR3 | hsa-mir-548aj-3p | -0.0190 | 0.712 |
| CELSR3 | hsa-mir-548ak | 0.0080 | 0.879 |
| CELSR3 | hsa-mir-548am-3p | 0.0970 | 0.062 |
| CELSR3 | hsa-mir-3156-3p | 0.0040 | 0.938 |
| CELSR3 | hsa-mir-3162-3p | 0.0160 | 0.757 |
| CELSR3 | hsa-mir-3691-3p | 0.0930 | 0.075 |
| CELSR3 | hsa-mir-4446-5p | 0.0770 | 0.139 |
| CELSR3 | hsa-mir-3973 | 0.0000 | 1.000 |
| CELSR3 | hsa-mir-4646-3p | -0.1870 | 0.000 |
| CELSR3 | hsa-mir-4691-5p | -0.0110 | 0.828 |
| CELSR3 | hsa-mir-4755-5p | 0.0290 | 0.581 |
| CELSR3 | hsa-mir-548ah-3p | 0.0140 | 0.788 |
| CELSR3 | hsa-mir-5006-3p | -0.0100 | 0.844 |
| CELSR3 | hsa-mir-548ap-5p | 0.0000 | 1.000 |
| CELSR3 | hsa-mir-548aq-5p | 0.0850 | 0.102 |
| CELSR3 | hsa-mir-548aq-3p | 0.0000 | 1.000 |
| CELSR3 | hsa-mir-548ar-5p | 0.0000 | 1.000 |
| CELSR3 | hsa-mir-548as-5p | 0.0000 | 1.000 |
| CELSR3 | hsa-mir-548au-5p | 0.0000 | 1.000 |
| CELSR3 | hsa-mir-5695 | 0.0860 | 0.098 |
| CELSR3 | hsa-mir-548h-3p | 0.0000 | 1.000 |
| CELSR3 | hsa-mir-548o-5p | 0.0000 | 1.000 |
| CELSR3 | hsa-mir-548am-5p | 0.0000 | 1.000 |
| CELSR3 | hsa-mir-548ay-5p | 0.0000 | 1.000 |
| CELSR3 | hsa-mir-6500-3p | -0.0590 | 0.259 |
| CELSR3 | hsa-mir-548j-3p | 0.0430 | 0.414 |
| CELSR3 | hsa-mir-6769a-5p | 0.0090 | 0.867 |
| CELSR3 | hsa-mir-6782-3p | 0.0260 | 0.619 |
| CELSR3 | hsa-mir-6792-3p | -0.0280 | 0.594 |
| CELSR3 | hsa-mir-6769b-5p | -0.0200 | 0.705 |
| CELSR3 | hsa-mir-6883-3p | 0.0390 | 0.449 |
| CELSR3 | hsa-mir-7110-3p | -0.0030 | 0.960 |
| CELSR3 | hsa-mir-8055 | 0.0000 | 1.000 |
| CELSR3 | hsa-mir-8081 | 0.0000 | 1.000 |
| CELSR3 | hsa-mir-548ad-5p | 0.0000 | 1.000 |
| CELSR3 | hsa-mir-548ae-5p | 0.0000 | 1.000 |
| CELSR3 | hsa-mir-548bb-5p | 0.0000 | 1.000 |
| CELSR3 | hsa-mir-548bb-3p | 0.0000 | 1.000 |
| CHEK1 | hsa-mir-15a-5p | 0.1280 | 0.014 |
| CHEK1 | hsa-mir-16-5p | 0.3130 | 0.000 |
| CHEK1 | hsa-mir-19a-3p | 0.1860 | 0.000 |
| CHEK1 | hsa-mir-19b-3p | 0.1440 | 0.006 |
| CHEK1 | hsa-mir-24-3p | 0.2070 | 0.000 |
| CHEK1 | hsa-mir-26a-5p | 0.0040 | 0.939 |
| CHEK1 | hsa-mir-92a-3p | 0.1750 | 0.001 |
| CHEK1 | hsa-mir-197-3p | 0.3450 | 0.000 |
| CHEK1 | hsa-mir-15b-5p | 0.4900 | 0.000 |
| CHEK1 | hsa-mir-195-5p | -0.1170 | 0.025 |
| CHEK1 | hsa-mir-99b-5p | 0.1970 | 0.000 |
| CHEK1 | hsa-mir-323a-3p | 0.2090 | 0.000 |
| CHEK1 | hsa-mir-424-5p | 0.0120 | 0.821 |
| CHEK1 | hsa-mir-329-3p | 0.1440 | 0.005 |
| CHEK1 | hsa-mir-410-3p | 0.1960 | 0.000 |
| CHEK1 | hsa-mir-193b-3p | -0.1210 | 0.020 |
| CHEK1 | hsa-mir-497-5p | -0.1370 | 0.008 |
| CHEK1 | hsa-mir-503-5p | 0.2300 | 0.000 |
| CHEK1 | hsa-mir-505-3p | -0.0390 | 0.451 |
| CHEK1 | hsa-mir-603 | -0.0230 | 0.659 |
| CHEK1 | hsa-mir-620 | 0.0000 | 1.000 |
| CHEK1 | hsa-mir-646 | 0.0000 | 1.000 |
| CHEK1 | hsa-mir-766-3p | 0.1410 | 0.007 |
| CHEK1 | hsa-mir-139-3p | -0.3890 | 0.000 |
| CHEK1 | hsa-mir-362-3p | 0.0250 | 0.626 |
| CHEK1 | hsa-mir-508-5p | 0.0360 | 0.490 |
| CHEK1 | hsa-mir-1226-3p | 0.1960 | 0.000 |
| CHEK1 | hsa-mir-1227-3p | 0.1260 | 0.015 |
| CHEK1 | hsa-mir-1270 | 0.3410 | 0.000 |
| CHEK1 | hsa-mir-466 | 0.0530 | 0.306 |
| CHEK1 | hsa-mir-4252 | 0.0000 | 1.000 |
| CHEK1 | hsa-mir-3941 | 0.0360 | 0.496 |
| CHEK1 | hsa-mir-4524a-5p | -0.0560 | 0.283 |
| CHEK1 | hsa-mir-4531 | 0.0000 | 1.000 |
| CHEK1 | hsa-mir-4672 | 0.0790 | 0.131 |
| CHEK1 | hsa-mir-4731-3p | 0.0320 | 0.544 |
| CHEK1 | hsa-mir-4733-3p | 0.0330 | 0.533 |
| CHEK1 | hsa-mir-4789-3p | -0.0110 | 0.838 |
| CHEK1 | hsa-mir-4793-3p | 0.2490 | 0.000 |
| CHEK1 | hsa-mir-4801 | 0.0000 | 1.000 |
| CHEK1 | hsa-mir-4524b-5p | 0.0000 | 1.000 |
| CHEK1 | hsa-mir-550b-2-5p | 0.1000 | 0.055 |
| CHEK1 | hsa-mir-1273g-3p | 0.0000 | 1.000 |
| CHEK1 | hsa-mir-6074 | 0.0000 | 1.000 |
| CHEK1 | hsa-mir-6083 | 0.0000 | 1.000 |
| CHEK1 | hsa-mir-6507-5p | 0.2020 | 0.000 |
| CHEK1 | hsa-mir-190a-3p | 0.1080 | 0.038 |
| CHEK1 | hsa-mir-383-3p | 0.0860 | 0.100 |
| CHEK1 | hsa-mir-513b-3p | 0.1170 | 0.025 |
| CHEK1 | hsa-mir-6838-5p | -0.0130 | 0.809 |
| CHEK1 | hsa-mir-7703 | 0.0960 | 0.065 |
| CHEK1 | hsa-mir-8485 | 0.0000 | 1.000 |
| GPSM2 | hsa-mir-17-3p | 0.1160 | 0.025 |
| GPSM2 | hsa-mir-192-5p | -0.1530 | 0.003 |
| GPSM2 | hsa-mir-215-5p | -0.0890 | 0.088 |
| GPSM2 | hsa-mir-122-5p | -0.3770 | 0.000 |
| GPSM2 | hsa-mir-373-3p | 0.2120 | 0.000 |
| GPSM2 | hsa-mir-378a-5p | -0.1210 | 0.020 |
| GPSM2 | hsa-mir-323a-3p | 0.1220 | 0.019 |
| GPSM2 | hsa-mir-564 | 0.0140 | 0.786 |
| GPSM2 | hsa-mir-569 | 0.0000 | 1.000 |
| GPSM2 | hsa-mir-32-3p | 0.1250 | 0.016 |
| GPSM2 | hsa-mir-143-5p | -0.0430 | 0.412 |
| GPSM2 | hsa-mir-103b | -0.0140 | 0.790 |
| GPSM2 | hsa-mir-3065-3p | -0.0630 | 0.229 |
| GPSM2 | hsa-mir-3652 | -0.0210 | 0.682 |
| GPSM2 | hsa-mir-3660 | 0.3070 | 0.000 |
| GPSM2 | hsa-mir-4430 | -0.0130 | 0.796 |
| GPSM2 | hsa-mir-3135b | 0.0000 | 1.000 |
| GPSM2 | hsa-mir-4526 | 0.1410 | 0.007 |
| GPSM2 | hsa-mir-4474-5p | -0.0540 | 0.299 |
| GPSM2 | hsa-mir-4704-3p | 0.0790 | 0.128 |
| GPSM2 | hsa-mir-5687 | 0.0560 | 0.279 |
| GPSM2 | hsa-mir-5693 | 0.0330 | 0.532 |
| GPSM2 | hsa-mir-5698 | 0.1830 | 0.000 |
| GPSM2 | hsa-mir-6499-3p | 0.0350 | 0.500 |
| GPSM2 | hsa-mir-215-3p | 0.1440 | 0.006 |
| GPSM2 | hsa-mir-383-3p | 0.0090 | 0.866 |
| GPSM2 | hsa-mir-504-3p | 0.1510 | 0.004 |
| GPSM2 | hsa-mir-891a-3p | -0.0310 | 0.554 |
| GPSM2 | hsa-mir-500b-3p | 0.2150 | 0.000 |
| GPSM2 | hsa-mir-6773-3p | -0.0130 | 0.807 |
| GPSM2 | hsa-mir-6854-5p | 0.1140 | 0.029 |
| GPSM2 | hsa-mir-6879-3p | 0.1300 | 0.012 |
| GPSM2 | hsa-mir-7705 | -0.0240 | 0.642 |
