# Supplementary material for: A Key mRNA-miRNA-lncRNA Competing Endogenous RNA Triple Sub-network Linked to Diagnosis and Prognosis of Hepatocellular Carcinoma
Source: Front Oncol. 2020 Mar 17;10:340. doi: 10.3389/fonc.2020.00340 (PMC7092636; doi:10.3389/fonc.2020.00340)
Supplement: Supplementary file 3 [file Table_3.docx]

**Table S3.** The correlation between miRNA-mRNA pairs determined by starBase database.

| mRNA | miRNA | R | P-value |
| --- | --- | --- | --- |
| hsa-mir-335-5p | CLEC3B | 0.0880 | 0.091 |
| hsa-mir-335-5p | DNASE1L3 | 0.0690 | 0.185 |
| hsa-mir-1236-3p | DNASE1L3 | -0.0790 | 0.131 |
| hsa-mir-1184 | DNASE1L3 | 0.0000 | 1.000 |
| hsa-mir-4691-5p | DNASE1L3 | -0.0560 | 0.279 |
| hsa-mir-4725-5p | DNASE1L3 | -0.0730 | 0.163 |
| hsa-mir-6749-3p | DNASE1L3 | -0.0800 | 0.124 |
| hsa-mir-6792-3p | DNASE1L3 | -0.0250 | 0.630 |
| hsa-mir-6801-3p | DNASE1L3 | -0.0410 | 0.432 |
| hsa-mir-6810-3p | DNASE1L3 | -0.0480 | 0.359 |
| hsa-mir-6812-3p | DNASE1L3 | -0.1140 | 0.028 |
| hsa-mir-6819-3p | DNASE1L3 | 0.0570 | 0.276 |
| hsa-mir-6877-3p | DNASE1L3 | -0.0260 | 0.621 |
| hsa-mir-7156-3p | DNASE1L3 | 0.0070 | 0.889 |
| hsa-mir-16-5p | KIF2C | 0.3500 | 0.000 |
| hsa-mir-20a-5p | KIF2C | 0.2650 | 0.000 |
| hsa-mir-92a-3p | KIF2C | 0.2870 | 0.000 |
| hsa-mir-101-3p | KIF2C | -0.3480 | 0.000 |
| hsa-mir-148a-3p | KIF2C | -0.2420 | 0.000 |
| hsa-mir-34a-5p | KIF2C | -0.2590 | 0.000 |
| hsa-mir-181a-5p | KIF2C | 0.1780 | 0.001 |
| hsa-mir-181b-5p | KIF2C | 0.2560 | 0.000 |
| hsa-mir-181c-5p | KIF2C | 0.1470 | 0.005 |
| hsa-mir-1-3p | KIF2C | -0.0860 | 0.100 |
| hsa-mir-124-3p | KIF2C | 0.0480 | 0.361 |
| hsa-mir-142-5p | KIF2C | 0.2190 | 0.000 |
| hsa-mir-152-3p | KIF2C | -0.1540 | 0.003 |
| hsa-mir-148b-3p | KIF2C | 0.3050 | 0.000 |
| hsa-mir-423-3p | KIF2C | 0.4600 | 0.000 |
| hsa-mir-494-3p | KIF2C | 0.0700 | 0.180 |
| hsa-mir-181d-5p | KIF2C | 0.1540 | 0.003 |
| hsa-mir-517-5p | KIF2C | 0.2310 | 0.000 |
| hsa-mir-559 | KIF2C | 0.0170 | 0.751 |
| hsa-mir-583 | KIF2C | 0.0000 | 1.000 |
| hsa-mir-186-3p | KIF2C | 0.0800 | 0.127 |
| hsa-mir-545-5p | KIF2C | 0.1010 | 0.051 |
| hsa-mir-548b-5p | KIF2C | 0.1430 | 0.006 |
| hsa-mir-548a-5p | KIF2C | 0.2130 | 0.000 |
| hsa-mir-548c-5p | KIF2C | 0.0000 | 1.000 |
| hsa-mir-548d-5p | KIF2C | -0.0020 | 0.977 |
| hsa-mir-548j-5p | KIF2C | 0.0930 | 0.073 |
| hsa-mir-548k | KIF2C | 0.2430 | 0.000 |
| hsa-mir-548l | KIF2C | 0.2030 | 0.000 |
| hsa-mir-1303 | KIF2C | 0.0180 | 0.723 |
| hsa-mir-1251-5p | KIF2C | 0.1650 | 0.001 |
| hsa-mir-548h-5p | KIF2C | 0.0000 | 1.000 |
| hsa-mir-548i | KIF2C | 0.0000 | 1.000 |
| hsa-mir-1321 | KIF2C | 0.0000 | 1.000 |
| hsa-mir-3154 | KIF2C | 0.0420 | 0.415 |
| hsa-mir-3162-5p | KIF2C | 0.0250 | 0.633 |
| hsa-mir-548w | KIF2C | 0.0420 | 0.417 |
| hsa-mir-4262 | KIF2C | 0.0000 | 1.000 |
| hsa-mir-4276 | KIF2C | 0.0000 | 1.000 |
| hsa-mir-4330 | KIF2C | 0.0000 | 1.000 |
| hsa-mir-3675-3p | KIF2C | 0.0000 | 1.000 |
| hsa-mir-548y | KIF2C | 0.3440 | 0.000 |
| hsa-mir-548ab | KIF2C | -0.0320 | 0.536 |
| hsa-mir-548ak | KIF2C | -0.0290 | 0.573 |
| hsa-mir-4484 | KIF2C | -0.0600 | 0.247 |
| hsa-mir-4524a-3p | KIF2C | -0.1930 | 0.000 |
| hsa-mir-3136-3p | KIF2C | 0.0490 | 0.346 |
| hsa-mir-4645-3p | KIF2C | 0.0870 | 0.095 |
| hsa-mir-4739 | KIF2C | 0.1010 | 0.051 |
| hsa-mir-4756-5p | KIF2C | 0.0000 | 1.000 |
| hsa-mir-548ap-5p | KIF2C | 0.0000 | 1.000 |
| hsa-mir-548aq-5p | KIF2C | 0.2780 | 0.000 |
| hsa-mir-548ar-5p | KIF2C | 0.0000 | 1.000 |
| hsa-mir-548as-5p | KIF2C | 0.0000 | 1.000 |
| hsa-mir-548au-5p | KIF2C | 0.0000 | 1.000 |
| hsa-mir-548av-5p | KIF2C | 0.0540 | 0.298 |
| hsa-mir-548o-5p | KIF2C | 0.0000 | 1.000 |
| hsa-mir-548am-5p | KIF2C | 0.0000 | 1.000 |
| hsa-mir-6075 | KIF2C | 0.0000 | 1.000 |
| hsa-mir-548ay-5p | KIF2C | 0.0000 | 1.000 |
| hsa-mir-6760-5p | KIF2C | 0.0110 | 0.837 |
| hsa-mir-6858-3p | KIF2C | 0.1410 | 0.007 |
| hsa-mir-7113-5p | KIF2C | 0.0600 | 0.253 |
| hsa-mir-7155-3p | KIF2C | 0.0740 | 0.158 |
| hsa-mir-8054 | KIF2C | 0.0000 | 1.000 |
| hsa-mir-548ad-5p | KIF2C | 0.0000 | 1.000 |
| hsa-mir-548ae-5p | KIF2C | 0.0000 | 1.000 |
| hsa-mir-548bb-5p | KIF2C | 0.0000 | 1.000 |
| hsa-let-7b-5p | PTTG1 | -0.1440 | 0.005 |
| hsa-mir-17-5p | PTTG1 | 0.3550 | 0.000 |
| hsa-mir-26a-5p | PTTG1 | -0.0690 | 0.184 |
| hsa-mir-186-5p | PTTG1 | 0.1990 | 0.000 |
| hsa-mir-320a | PTTG1 | 0.0860 | 0.097 |
| hsa-mir-655-3p | PTTG1 | 0.1510 | 0.004 |
| hsa-mir-146a-3p | PTTG1 | 0.2080 | 0.000 |
| hsa-mir-423-5p | PTTG1 | 0.3240 | 0.000 |
| hsa-mir-16-5p | UBE2S | 0.2390 | 0.000 |
| hsa-mir-17-5p | UBE2S | 0.2820 | 0.000 |
| hsa-mir-34a-5p | UBE2S | -0.2040 | 0.000 |
| hsa-mir-149-5p | UBE2S | 0.2420 | 0.000 |
| hsa-mir-324-5p | UBE2S | 0.3060 | 0.000 |
| hsa-mir-484 | UBE2S | 0.2150 | 0.000 |
| hsa-mir-486-5p | UBE2S | -0.1020 | 0.051 |
| hsa-mir-571 | UBE2S | -0.0090 | 0.859 |
| hsa-mir-32-3p | UBE2S | 0.1940 | 0.000 |
| hsa-mir-92a-1-5p | UBE2S | 0.2360 | 0.000 |
| hsa-mir-149-3p | UBE2S | 0.1680 | 0.001 |
| hsa-mir-377-5p | UBE2S | 0.0550 | 0.291 |
| hsa-mir-615-5p | UBE2S | 0.0420 | 0.424 |
| hsa-mir-1303 | UBE2S | 0.0250 | 0.635 |
| hsa-mir-1321 | UBE2S | 0.0000 | 1.000 |
| hsa-mir-1825 | UBE2S | 0.0140 | 0.792 |
| hsa-mir-3170 | UBE2S | 0.2100 | 0.000 |
| hsa-mir-3202 | UBE2S | -0.0500 | 0.337 |
| hsa-mir-4313 | UBE2S | 0.0000 | 1.000 |
| hsa-mir-4419a | UBE2S | 0.0000 | 1.000 |
| hsa-mir-4443 | UBE2S | 0.0900 | 0.084 |
| hsa-mir-4510 | UBE2S | -0.1120 | 0.032 |
| hsa-mir-4524a-3p | UBE2S | -0.1970 | 0.000 |
| hsa-mir-4533 | UBE2S | 0.1450 | 0.005 |
| hsa-mir-4728-5p | UBE2S | 0.0330 | 0.526 |
| hsa-mir-4739 | UBE2S | 0.0820 | 0.115 |
| hsa-mir-371b-3p | UBE2S | 0.1020 | 0.050 |
| hsa-mir-4756-5p | UBE2S | 0.0000 | 1.000 |
| hsa-mir-4768-3p | UBE2S | 0.0460 | 0.377 |
| hsa-mir-4797-5p | UBE2S | -0.0050 | 0.928 |
| hsa-mir-5572 | UBE2S | 0.0490 | 0.348 |
| hsa-mir-5692a | UBE2S | -0.0290 | 0.575 |
| hsa-mir-3190-3p | UBE2S | -0.0140 | 0.786 |
| hsa-mir-6086 | UBE2S | 0.0000 | 1.000 |
| hsa-mir-6127 | UBE2S | 0.0000 | 1.000 |
| hsa-mir-6129 | UBE2S | -0.0470 | 0.365 |
| hsa-mir-6130 | UBE2S | -0.0010 | 0.980 |
| hsa-mir-6133 | UBE2S | 0.0000 | 1.000 |
| hsa-mir-6515-5p | UBE2S | 0.0240 | 0.646 |
| hsa-mir-6737-5p | UBE2S | 0.0980 | 0.060 |
| hsa-mir-6785-5p | UBE2S | 0.0730 | 0.158 |
| hsa-mir-6797-5p | UBE2S | -0.0170 | 0.747 |
| hsa-mir-6812-5p | UBE2S | 0.1780 | 0.001 |
| hsa-mir-6819-5p | UBE2S | 0.1460 | 0.005 |
| hsa-mir-6855-5p | UBE2S | 0.0900 | 0.084 |
| hsa-mir-6883-5p | UBE2S | -0.0380 | 0.465 |
| hsa-mir-7704 | UBE2S | 0.0030 | 0.954 |
| hsa-mir-7848-3p | UBE2S | 0.0390 | 0.578 |
| hsa-mir-1249-5p | UBE2S | 0.2280 | 0.000 |
| hsa-let-7b-5p | XPO5 | -0.1090 | 0.035 |
| hsa-mir-216a-5p | XPO5 | -0.1480 | 0.004 |
| hsa-mir-122-5p | XPO5 | -0.1860 | 0.000 |
| hsa-mir-425-3p | XPO5 | 0.2350 | 0.000 |
| hsa-mir-92b-3p | XPO5 | 0.1240 | 0.017 |
| hsa-mir-143-5p | XPO5 | -0.1010 | 0.053 |
| hsa-mir-509-5p | XPO5 | 0.0080 | 0.873 |
| hsa-mir-744-3p | XPO5 | 0.2890 | 0.000 |
| hsa-mir-509-3-5p | XPO5 | 0.0660 | 0.208 |
| hsa-mir-1301-3p | XPO5 | 0.2500 | 0.000 |
| hsa-mir-1184 | XPO5 | 0.0000 | 1.000 |
| hsa-mir-3652 | XPO5 | -0.0190 | 0.719 |
| hsa-mir-4418 | XPO5 | 0.0700 | 0.180 |
| hsa-mir-4430 | XPO5 | -0.0640 | 0.218 |
| hsa-mir-3135b | XPO5 | 0.0000 | 1.000 |
| hsa-mir-4423-5p | XPO5 | -0.0070 | 0.895 |
| hsa-mir-4660 | XPO5 | 0.1270 | 0.015 |
| hsa-mir-5047 | XPO5 | 0.0250 | 0.635 |
| hsa-mir-5698 | XPO5 | 0.0630 | 0.228 |
| hsa-mir-1304-3p | XPO5 | 0.1990 | 0.000 |
| hsa-mir-6499-3p | XPO5 | -0.0440 | 0.398 |
| hsa-mir-6501-5p | XPO5 | -0.0040 | 0.943 |
| hsa-mir-504-3p | XPO5 | 0.0250 | 0.632 |
| hsa-mir-6736-3p | XPO5 | 0.0740 | 0.157 |
| hsa-mir-6787-3p | XPO5 | 0.0430 | 0.413 |
| hsa-mir-6890-3p | XPO5 | 0.0890 | 0.086 |
