# Supplementary figures and images for: A Key mRNA-miRNA-lncRNA Competing Endogenous RNA Triple Sub-network Linked to Diagnosis and Prognosis of Hepatocellular Carcinoma
Source: Front Oncol. 2020 Mar 17;10:340. doi: 10.3389/fonc.2020.00340 (PMC7092636; doi:10.3389/fonc.2020.00340)

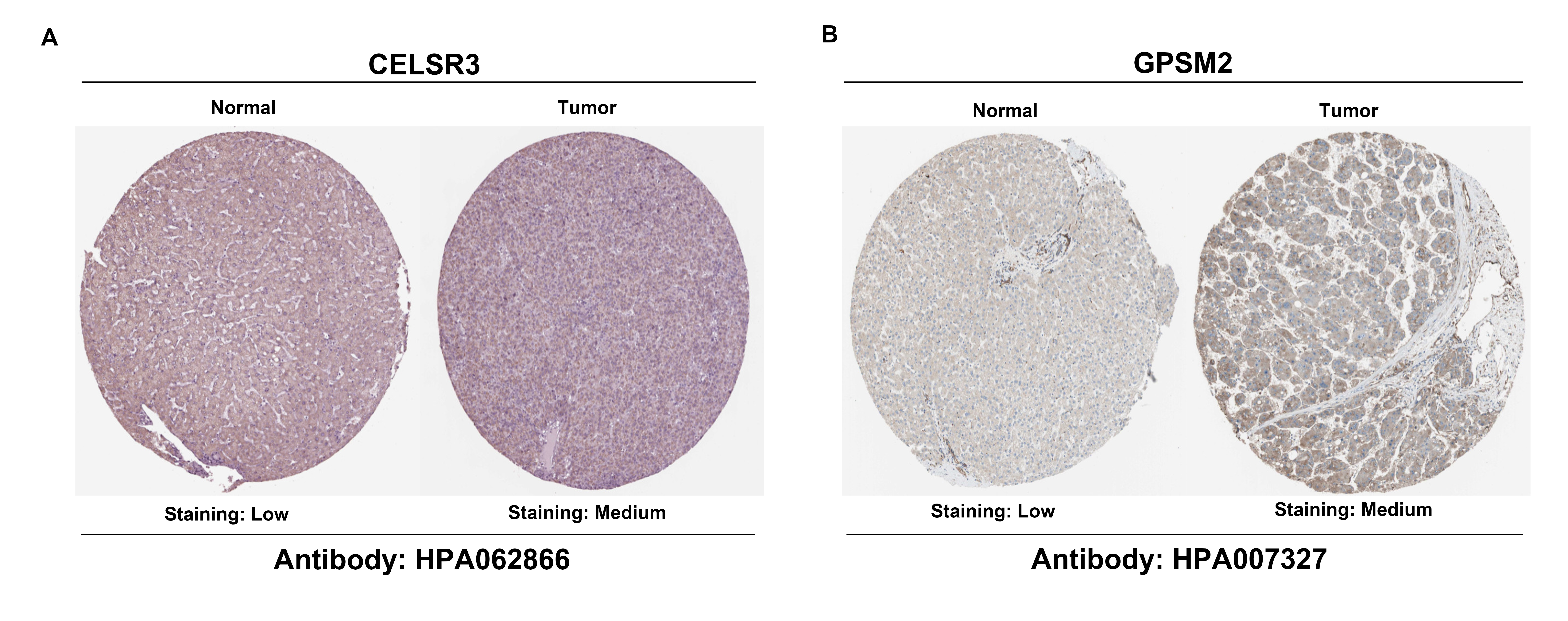

Supplement: Supplementary file 4 [file Image_1.JPEG]
